# Contribution of Transcriptomics to Systems-Level Understanding of Methanogenic *Archaea*

**DOI:** 10.1155/2013/586369

**Published:** 2013-02-27

**Authors:** Patrick D. Browne, Hinsby Cadillo-Quiroz

**Affiliations:** ^1^School of Life Sciences, Arizona State University, 427 E. Tyler Mall, Tempe, AZ 85287, USA; ^2^Biodesign Institute, Arizona State University, 427 E. Tyler Mall, Tempe, AZ 85287, USA

## Abstract

Methane-producing *Archaea* are of interest due to their contribution to atmospheric change and for their roles in technological applications including waste treatment and biofuel production. Although restricted to anaerobic environments, methanogens are found in a wide variety of habitats, where they commonly live in syntrophic relationships with bacterial partners. Owing to tight thermodynamic constraints of methanogenesis alone or in syntrophic metabolism, methanogens must carefully regulate their catabolic pathways including the regulation of RNA transcripts. The transcriptome is a dynamic and important control point in microbial systems. This paper assesses the impact of mRNA (transcriptome) studies on the understanding of methanogenesis with special consideration given to how methanogenesis is regulated to cope with nutrient limitation, environmental variability, and interactions with syntrophic partners. In comparison with traditional microarray-based transcriptome analyses, next-generation high-throughput RNA sequencing is greatly advantageous in assessing transcription start sites, the extent of 5′ untranslated regions, operonic structure, and the presence of small RNAs. We are still in the early stages of understanding RNA regulation but it is already clear that determinants beyond transcript abundance are highly relevant to the lifestyles of methanogens, requiring further study.

## 1. Introduction

Methane- (CH_4_-) producing *Archaea* occupy an important position in the global carbon cycle and in atmospheric change by performing the final steps of biomass degradation in anaerobic systems, and releasing significant amounts of CH_4_ to the atmosphere every year [1]. Also, methanogenic *Archaea* are of interest due to their role in anaerobic degradation including waste treatment, biogenic gas production from coal, and other substrates that have potential for CH_4_ to be harvested for use as a fuel. Therefore, considerable environmental and economic benefits may come from understanding biological CH_4_ production.

**Figure 1:**
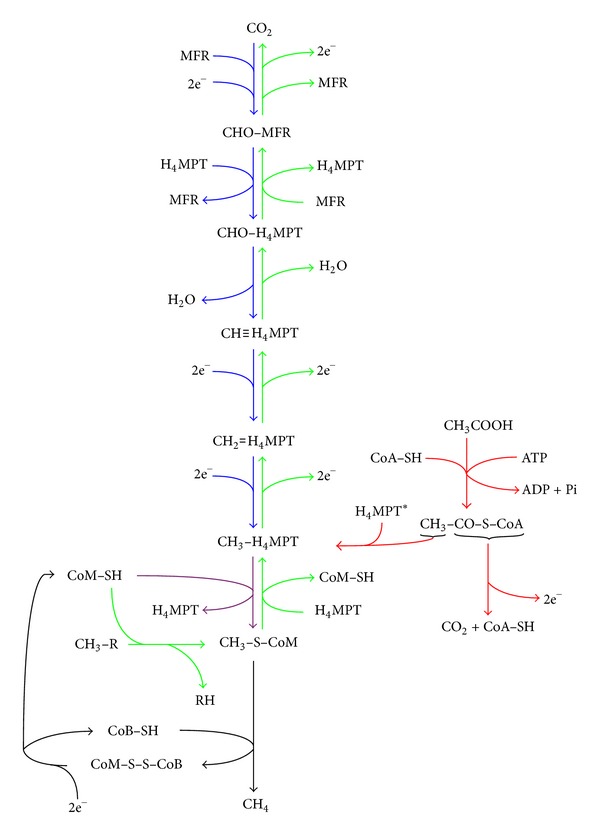
Overview of the three major known methanogenic pathways in Archaea. Color coding indicates the steps common to all three types (black), unique to the methylotrophic pathway (green), unique to the hydrogenotrophic (or CO_2_ reducing) pathway (blue), unique to the aceticlastic pathway (red), and shared between hydrogenotrophic and aceticlastic methanogenesis (purple). 2e^−^ represents reducing equivalents, produced or consumed during each reaction. MFR: methanofuran; H_4_MPT: tetrahydromethanopterin; CoM-SH: coenzyme M; CoB-SH: coenzyme B; CoA-SH: coenzyme A; CoM-S-S-CoB: heterodisulfide of coenzyme M and coenzyme B; ATP: adenosine triphosphate; R: ligand bound to methylated compound that serves as substrate for methylotrophic methanogenesis. *Tetrahydrosarcinapterin is a functional analogue of H_4_MPT found in the Methanosarcinales order of methanogens.

In terms of physiology, three major, partially overlapping, methanogenesis pathways are recognized: (i) methanogenesis from carbon dioxide (CO_2_) reduction with hydrogen (H_2_) (hydrogenotrophic pathway), (ii) methanogenesis from methylated compounds such as methanol and methylated amines (methylotrophic pathway), and (iii) methanogenesis from acetate cleavage (aceticlastic pathway). The biochemistry of methanogenesis was reviewed elsewhere [2–4] and is summarized in Figure 1. The only known biological producers of CH_4_ are a diverse range of anaerobic *Archaea* within the Euryarchaeota phylum including the following orders: Methanobacteriales, Methanococcales, Methanocellales, Methanosarcinales, Methanomicrobiales, and Methanopyrales [3, 5] and the recently proposed Methanoplasmatales [6]. Members of the Methanosarcinales order have the widest substrate range where all three major methanogenic pathways are represented, while the other orders generally perform methanogenesis only via CO_2_ reduction [3, 4].

Because of a lower bioenergetic yield compared to other reactions used by microbial groups, methanogens generally thrive in environments or conditions lacking terminal electron acceptors other than CO_2_ [7]. Methanogens are found in a diverse range of habitats including wetlands, rice paddies, fresh and marine water sediments, digestive tracts of ruminants and termites, anaerobic waste digesters, and geothermal vents. These habitats feature a broad range of environmental conditions with strong variation of temperature, salinity, nutrient availability, and pH. Methanogens generally grow syntrophically with fermentative bacteria which produce methanogenic substrates. Careful regulation of metabolism on the part of both syntrophic partners is required owing to the tight thermodynamic constraints of conversion of biomass to CH_4_.

In order to understand the activity of methanogenic *Archaea* and their contribution in different ecosystems, it is important to address how methanogenesis is regulated at the genetic and cellular systems levels and how these regulations impact upon the adaptation of methanogens to their environments and their interactions with their syntrophic partners. Gene expression and regulation in methanogenic *Archaea* has not been completely characterized and is thus an important missing link in the understanding of these organisms.

RNA plays a central role in gene expression as an intermediate between genes and proteins, as an adapter during translation, and as a component in ribosomes. Transcriptomic approaches aim to characterize the RNA content of a sample of interest, facilitating estimations of gene expression levels and identification of differentially expressed genes between different treatments. This paper aims to discuss the ways in which transcriptome analyses have broadened the understanding of the regulation of methanogenesis and the ecology of methanogens. To this end, the effects of reducing power supply, the significance of isofunctional enzymes, and the use of alternate energy conserving pathways during methanogenesis will be major points of discussion. Transcriptome-based systems-level assessments of how methanogens adapt to stresses and limitations in their environments and niches are also reviewed. Furthermore, this paper will summarize the current body of knowledge about RNA regulation in methanogenesis and how emerging technologies are advancing research in this area.

## 2. Methanogenesis Is Fine Tuned to Meet Niche Requirements

### 2.1. Response of Methanogenic Pathways to Substrate Supply

Methanogenesis-related mRNAs are amongst the most highly abundant in methanogens. For example, it has been shown that 33 of the 100 most highly abundant mRNAs detected in *Methanosarcina barkeri* DSM 804 grown on methanol are related to methanogenesis [8]. However, for reasons of efficiency, methanogenesis-related mRNAs are not constitutively present at high levels, but rather their levels are carefully regulated in order to facilitate optimization of metabolism relative to growth conditions. A common strategy of gene regulation is transcriptional upregulation only in the presence of the substrate of the gene product. This is especially important in the metabolically diverse Methanosarcinales order. When methanol-grown *Methanosarcina mazei* or *Methanosarcina acetivorans* (both members of the Methanosarcinales) were compared with their acetate-grown counterparts, transcriptome analyses showed that the genes specific to the methylotrophic and aceticlastic methanogenic pathways were regulated in substrate-dependent manners [9, 10]. Transcript levels of genes encoding different isozymes of methanol-specific methanol transferase and corrinoid proteins are subject to transcriptional control by promoter activity and posttranscriptional control by means of differential transcript stabilities, as indicated by transcriptional fusions, translational fusions, and qRT-PCR [11]. Transcriptome sequencing revealed that a regulator, known as MreA, was responsible for regulating 280 genes, either directly or indirectly, when *M. acetivorans* was grown with acetate [12]. DNA-binding experiments reported with this transcriptome data set indicated that MreA can directly upregulate transcription of acetate catabolism genes and directly downregulate methanol catabolism genes [12].

The pathways of methanogenesis from methanol and from methylamines are identical apart from the use of substrate-specific methyltransferases and corrinoid proteins involved in the initial transfer of the methyl group from the substrate to coenzyme M (CoM-SH) (Figure 1). The utilization of trimethylamine (TMA) as a methanogenic substrate is complicated further since it is degraded to two other methanogenic substrates, dimethylamine (DMA) and monomethylamine (MMA), during its conversion into CH_4_, CO_2_, and ammonia. Each of TMA, DMA, and MMA also requires substrate-specific methyltransferases and corrinoid proteins, each of which is encoded by two to three homologous genes in *M. mazei*. Microarray-based transcriptome analysis indicated that the mRNA levels of 72 genes were different in TMA-grown versus methanol-grown *M. mazei* [13]. None of the differences occurred in the core methanogenesis pathway or in energy conservation. The major substrate dependent differences occurred in the mRNA levels of the specific methyltransferases, with different homologues regulated to different extents. Monitoring of transcript levels of the methyltransferase and corrinoid genes via qRT-PCR in conjunction with chemical analysis of the culture medium indicated that *M. mazei* features a gene expression program to firstly utilize TMA followed by DMA and finally MMA, as would be expected. Although it is expected that genes involved in TMA degradation would be upregulated in the presence of TMA, it is vital, nonetheless, to understand the dynamics of primary metabolism and these results exemplified the validity of this approach. One of the strengths of global transcriptome analyses (and other global analyses) is the identification of non-intuitive cellular responses. For example, TMA grown *M. mazei* also evidenced higher transcript levels of genes involved in aromatic amino acid biosynthesis and ether lipid synthesis [13], the latter possibly being a response to combat the ability of TMA to depolarize the cell membrane.

Thermodynamically, hydrogenotrophic and methylotrophic methanogenesis are more favorable than aceticlastic methanogenesis. Consequently, acetate-grown *Methanosarcina* spp. grow slower than their methanol- or TMA-grown counterparts and utilize methylated compounds in preference to acetate [14]. The precise mechanisms of energy conservation operating in the various methanogenesis pathways are still a topic of active investigation. However key differences in energy conservation exist between the hydrogenotrophic, methylotrophic, and aceticlastic methanogenesis pathways. In the Methanosarcinales order, the use of a cytochrome-containing, membrane-bound HdrED type heterodisulfide reductase conserves some of the free energy of coenzyme M-coenzyme B heterodisulfide (CoM-S-S-CoB) reduction through the generation of a transmembrane proton gradient [2, 4]. On the other hand, hydrogenotrophic methanogens outside of the Methanosarcinales order utilize a soluble HdrABC type heterodisulfide reductase that couples the exergonic heterodisulfide reduction with the endergonic reduction of CO_2_ during the formyl-methanofuran (formyl-MFR) formation step [15].

The trafficking of electrons to the different Hdr complexes gives rise to key differences in energy conservation between the different methanogenic pathways [4]. Microarray-based transcriptome analyses of methanogens grown with either acetate or a methylotrophic substrate are shedding light on some of these differences [9, 10, 16]. The higher expression of Ech hydrogenase, an A_1_A_0_-type ATP synthase, and genes coding for flavoproteins and ferredoxins in acetate-grown cells, as opposed to methanol-grown cells, indicate that these components are part of a different pathway of electron trafficking and energy conservation during aceticlastic methanogenesis in *M. mazei *[9]. In comparison with the wild type strain, an *ech *mutant of *M. mazei* had a reduced growth rate and yield whilst utilizing TMA and was completely unable to grow using acetate as a sole methanogenic substrate [17]. The role of Ech hydrogenase is thought to be in accepting electrons from reduced ferredoxin, contributing to the transmembrane proton gradient and producing H_2_, the reducing power of which will eventually be used to reduce CoM-S-S-CoB [17, 18]. Higher transcript levels of the *M. mazei* HdrABC complex were reported in nitrogen-fixing conditions versus nitrogen- (ammonium) sufficient conditions in the simultaneous presence of methanol and acetate [19]. The biological significance of this phenomenon is unclear but it may be an adaptation to supply the energy and reducing equivalents necessary for nitrogen fixation.

In contrast to *M. mazei*, transcriptome analyses indicated that *M. acetivorans* possesses a different pathway that does not utilize an Ech hydrogenase, for electron flow and energy conservation associated with the reduction of CoM-S-S-CoB during aceticlastic methanogenesis [10, 16]. In this case, it was proposed that reduced ferredoxin donates its electrons to HdrED via the membrane-bound Rnf complex, with methanophenazine being a common electron trafficking intermediate. In this alternate pathway, energy is conserved through the generation of transmembrane ion gradients without using H_2_ as an intermediate. A freshwater *Methanosarcina* spp. also utilized H_2_ as an intermediate during aceticlastic methanogenesis, much like *M. mazei* and in contrast to the marine isolate *M. acetivorans* [18]. Avoiding the use of H_2_ as an intermediate during aceticlastic methanogenesis represents a competitive adaption to marine environments [18], where sulfate concentrations are relatively high (>20 mM), and competition for H_2_ by sulfate reducers is consequently higher than that in freshwater environments [20].

Although HdrED plays roles in both methylotrophic and aceticlastic methanogenesis, these two pathways employ very different energy conservation strategies. Transcript and mutant analyses indicate that the oxidation of reduced F_420_ by the membrane-bound F_420_ dehydrogenase (Fpo) plays a major role in electron trafficking in methylotrophic methanogenesis [16, 21]. Furthermore, the HdrABC complex plays a role in methylotrophic methanogenesis but not in aceticlastic methanogenesis [22]. Transcriptome analysis suggested that, in the absence of HdrABC, methanol-grown *M. acetivorans* is partially deficient in the ability to reduce CoM-S-S-CoB [22]. It has been suggested that electrons from formyl-MFR are donated to the HdrABC type heterodisulfide reductase, although this possibility remains untested [16].

**Figure 2:**
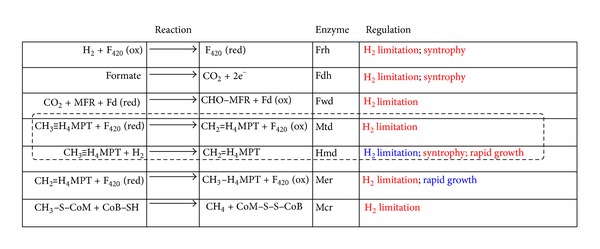
Observed regulatory patterns in hydrogenotrophic methanogenesis. Regulation steps for hydrogenotrophic methanogenesis are summarized from published sources discussed in this paper. Conditions, (syntrophic interaction or H_2_ limitation) causing upregulation and downregulation of enzyme transcript levels are indicated in red and blue, respectively. Abbreviations are as per Figure 1 with the addition of the following: F_420_: coenzyme F_420_; Fd: ferredoxin; Frh: F_420_-reducing hydrogenase; Fdh: formate dehydrogenase; Fwd: formyl-MFR dehydrogenase; Mtd: F_420_-dependent methylene-H_4_MPT dehydrogenase; Hmd: H_2_-dependent methylene-H_4_MPT dehydrogenase; Mer: F_420_-dependent methylene-H_4_MPT reductase; Mcr: methylcoenzyme M reductase. (The dotted lined box highlights two isofunctional enzymes oppositely regulated by H_2_ limitation).

In the CO_2_-reducing methanogenic pathway, some methanogenic steps are catalyzed by isofunctional enzymes where the same reduction step is coupled to the oxidation of a different electron donor. For example, the reduction of methenyl-tetrahydromethanopterin (methenyl-H_4_MPT) to methylene-H_4_MPT is catalyzed by both the F_420_-dependent methylene-H_4_MPT dehydrogenase (Mtd) and the H_2_-dependent methylene-H_4_MPT dehydrogenase (Hmd). These steps are often regulated in response to the supply of H_2_ or formate (Figure 2). For instance, in *Methanothermobacter thermautotrophicus*, microarray-based transcriptome analysis revealed that mRNA levels of F_420_-related targets (F_420_ reducing hydrogenase (*frh*), *mtd*, F_420_-dependent methylene-H_4_MPT reductase (*mer*), and methyl-CoM reductase (*mcr*)) were higher in H_2_-limited versus H_2_-sufficient conditions [23]. The upregulation of F_420_-related targets indicates that *M. thermautotrophicus* attempts to scavenge and use H_2_ more efficiently under conditions of H_2_ limitation where ferredoxin reduction is less thermodynamically favorable. Similarly, it was shown that genes related to F_420_ redox reactions in the hydrogenotrophic pathway of *Methanococcus maripaludis* were upregulated in response to H_2_ limitation [24]. Also, another study found that formate dehydrogenase (*fdh*) was upregulated during H_2_ limitation [25]. Interestingly, *hmd* showed higher levels of mRNA abundance under more rapid growth rate with H_2_ limitation, while growth rate did not regulate mRNA levels of *mtd* [25]. Hmd utilizes H_2_ directly with low affinity while Mtd uses reduced F_420_ as the electron donor (Figure 2). The preferential use of Mtd over Hmd at low H_2_ availability was also observed in a proteomics study, supporting the trends seen in the transcriptome analyses [26]. It has been suggested that Hmd and Mtd can act cyclically and produce H_2_ when, for example, reduced F_420_ is produced during formate oxidation [27]. This cyclical nature of Hmd and Mtd suggests that their regulation may be tied in with balancing the pools of oxidized and reduced F_420_ and to maintain a suitable H_2_ pool for ferredoxin reduction in order to optimize energy producing metabolism. However, in making such assertions from transcriptomics or proteomics data, it must be borne in mind that posttranscriptional levels of regulation of primary metabolism (e.g., allosterism) are very common, and biochemical studies are ultimately required to assess such hypotheses as mentioned previously.

Other methanogen optimizations in response to increased growth rate were the moderately increased mRNA levels of the genes for formyl-MFR:H_4_MPT methyltransferase and methenyl-H_4_MPT cyclohydrolase and moderately decreased mRNA levels of genes for heterodisulfide reductase [25]. It was shown, via a transcriptome sequencing approach in *Methanobrevibacter smithii*, that some components of the hydrogenotrophic pathway have strain-specific differences in mRNA levels [28]. These occurred for the *mtd* and *mer* genes and also for genes encoding components for an ABC-type cobalt transporter. Cobalt is an important part of corrinoid cofactors in methanogenesis-related enzymes, such as methyl-H_4_MPT-CoM methyltransferase (Mtr). These specific differences in methanogenesis pathways of *M. smithii* strains perhaps represent adaptations to various microhabitats within the heterogeneous human gastrointestinal tract, where *M. smithii* strains are the dominant *Archaea* [28].

Evidence for tight regulation of hydrogenotrophic methanogenesis was also demonstrated during syntrophic growth. Higher transcript levels of genes encoding H_2_-dependent methanogenesis-related targets were evident in *M. maripaludis* when grown in coculture with *Desulfovibrio vulgaris* than when the methanogen was grown alone under H_2_ limitation [29]. This indicates that *M. maripaludis* regulates its methanogenic pathway in order to facilitate syntrophy with *D. vulgaris* where the thermodynamics of both fermentative and methanogenic catabolisms must be carefully balanced. *M. thermautotrophicus* has two isofunctional Mcr enzymes, MRI and MRII. Northern blot analysis and proteomics show that both are expressed when *M. thermautotrophicus *is grown in pure culture in H_2_-sufficient conditions while only MRI was expressed during coculture with *Syntrophothermus lipocalidus* where H_2_ partial pressures were between 20 and 80 Pa [30].

### 2.2. Response of Methanogens to Environmental Perturbations

Transcriptome analyses of methanogens are useful not only for studying the process of methanogenesis but also for understanding the physiology and ecological adaptations of methanogens to their environments. Methanogens exhibit general adaptations to different growth rates that are brought about by various stresses such as nutrient limitation [9, 10, 19], heat stress, and oxidative stress [31] that generally involve downregulation of the translation apparatus in response to reduced cellular demands at low growth rates [24]. However, methanogens do not feature a universal stress response [23].

Nutrient limitation is very relevant when characterizing the ability of an organism to survive and adapt to changes in its environment. Transcriptomics indicated that *M. mazei* increased transcript levels of the core genes of nitrogen metabolism (nitrogenase, ammonium transporters, and glutamine synthetase) and several other genes including a cobalt transporter and genes with potential regulatory functions [19]. Similarly, a proteomics investigation revealed that *M. maripaludis* increased protein levels of nitrogenase, glutamine synthetase, ammonia transporters, and a nitrogen sensor/regulator in response to nitrogen limitation [26]. As mentioned previously, *M. mazei* showed higher levels of transcripts for HdrABC in nitrogen-fixing conditions, possibly related to increased demand for reducing equivalents for N_2_ reduction. Interestingly, a homologue of Hmd (HmdII) also increased in abundance in *M. maripaludis* in response to nitrogen-limiting conditions. Whether or not the alteration in HmdII plays any role in altering the dynamics of reducing equivalent pools remains unknown. For *M. mazei*, it was possible to predict a DNA motif involved in nitrogen responsiveness, and in the case of *M. maripaludis*, it was possible expand to upon the range of nitrogen responsive genes for a previously known motif. This is a good demonstration of the potential for omics technologies to generate hypotheses for further investigations, exemplified by investigations into DNA binding by the nitrogen-related transcriptional repressor, known as NrpR [32]. Complementary transcriptomics and proteomics studies identified three phosphate responsive phosphate transporters and a phosphate responsive putative phosphate transport regulator in *M. maripaludis* [24, 26]. Carbon monoxide dehydrogenase/acetyl coenzyme A synthase also appears to be downregulated during phosphate limitation [26].

Adaptation to temperature stress often involves alteration of cell surface components and the synthesis of molecular chaperones. In *M. barkeri*, heat shock resulted in the alteration of the levels of 168 transcripts [31]. Most notable was the increase in transcript abundance of Hsp70 and Hsp60. In *M. thermautotrophicus*, Hsp70 (but not Hsp60) was also responsive to heat stress and other stresses, such as oxidative stress and high pH [23]. The synthesis of chaperones was an adaptation to heat and cold stress by *Methanococcoides burtonii* [33, 34]. *M. burtonii* also showed increased levels of transcripts for RNA-binding proteins in response to cold stress, and it was suggested that this was an adaptive mechanism whereby RNA was maintained in a state suitable for translation [33, 34]. Transcriptome and proteome analyses of *M. burtonii* both indicated that remodeling of the cell surface was an important adaptation to heat and cold stress [33, 34]. Interestingly, 7 of the 10 most differentially abundant transcripts in response to cold stress for *M. burtonii *encoded genes of unknown function [33]. This suggests that *M. burtonii* also utilizes novel cold adaptive mechanisms.

Sodium stress is an important factor to consider particularly for methanogens inhabiting a wide range of sites including freshwater, marine, or transitional environments. In *M. mazei*, the most highly differentially expressed gene during sodium chloride stress was a hypothetical protein with no known homologues outside of the *Methanosarcina *genus [35]. The second most differentially expressed gene encoded a putative hypothetical protein. This indicates that some novel salt adaptive mechanisms may be present in *M. mazei*. However, conventional salt adaptive mechanisms were also evident in *M. mazei* with the upregulation of transcript levels related to solute transport and biosynthesis and Na^+^ export in response to high sodium chloride concentrations [35]. Interestingly, among nonmarine methanogens, their sensitivity to sodium levels is rather variable from highly to less sensitive [36]. The role of such a variable response has not been systematically evaluated but can play a role in the abundance of methanogenic groups in oligotrophic and minerotrophic environments as seen among different types of wetlands (e.g., [37]).

Methanogens grow under strictly anaerobic conditions and are highly sensitive to oxidative stress. Superoxide dismutase and catalase activities are typical components of oxidative stress responses. The activities of catalase and superoxide dismutase and the levels of their corresponding mRNAs were shown to be altered in *M. barkeri* in response to the oxidative stress inducing agents paraquat (*N*,*N *′-dimethyl-4,4′-bipyridinium dichloride) and hydrogen peroxide [38]. However, air exposure did not result in the upregulation of transcript levels of superoxide dismutase, catalase, or other nonspecific peroxidases in *M. barkeri* [31]. Rather, exposure of *M. barkeri* to air resulted in widespread changes in gene expression including upregulation of transposases and the downregulation of genes related to translation functions, amino acid transporters, energy metabolism, and signal transduction [31]. The oxidative stress response (induced by exposure to air) of *M. barkeri*, *Methanospirillum hungatei*, and two syntrophic bacteria, *D. vulgaris* and *Syntrophobacter fumaroxidans*, was studied in a defined coculture via a multispecies microarray transcriptome analysis approach [39]. In coculture, *M. barkeri* responded in a similar fashion as it did in pure culture with the downregulation of energy production and methanogenesis. *M. hungatei* responded to oxidative stress by upregulating thioredoxin and a heat shock protein (Hsp20) indicating that it deals with reactive oxygen species directly and attempts to protect its proteins from the effects of oxidative damage. In *M. thermautotrophicus*, an operon encoding a superoxide dismutase gene and other antioxidant enzymes did not alter in transcript level after exposure to hydrogen peroxide [23]. However, *M. thermautotrophicus* responded to hydrogen peroxide stress much like *M. barkeri* responded to air exposure, with the alteration of large numbers of transcript levels for processes related to translation, energy metabolism, amino acid transporters and metabolism, and coenzyme transport and metabolism [23]. From the above, it is clear that oxidative stress responses are varied, rather than conserved, between different methanogens. Given the extremely low oxygen tolerances typical of methanogens, it is not surprising that the most conserved aspect of oxidative stress responses is the downregulation of growth and metabolism, indicating that methanogens attempt to merely survive brief exposures to oxidative conditions.

### 2.3. Understanding Ecological Interaction through Transcripts

The regulations of the methanogenic pathways of *M. maripaludis* [29] and *M. thermautotrophicus* [30] in pure culture versus coculture were discussed before (see “Section 2.1”). Here we discuss other facets of these syntrophic interactions. *M. maripaludis* responded to syntrophic growth with *D. vulgaris* by downregulating transcripts associated with biosynthetic functions such as CO_2_ fixation [29]. This may seem counterintuitive since acetate was provided as a carbon source, in place of lactate, during monoculture, and suggests that syntrophically grown *M. maripaludis* may have received an assimilable carbon source from *D. vulgaris*. Evidence of transfer of alanine from *D. vulgaris* to *M. maripaludis* and an increase in transcript abundance of *M. maripaludis* alanine dehydrogenase suggests that alanine may be used by *M. maripaludis* as a carbon and nitrogen source during coculture [29]. The fact that alanine dehydrogenase activity produces reducing equivalents suggests the occurrence of a novel interspecies electron transfer mechanism. Generally, electron transfer between methanogens and their syntrophic partners involves formate and H_2_ [40, 41]. Interestingly, two seemingly isofunctional enzymes, the energy conserving hydrogenases Eha and Ehb, seemed to have opposite regulation, with Eha being upregulated and Ehb being down-regulated during coculture of *M. maripaludis* [29]. However, it was previously shown that Ehb functions in anabolism, while Eha plays a role in energy-generating metabolism [42]. This suggests that methanogens must carefully regulate both their growth and their metabolism in order to optimize the thermodynamics associated with the metabolism of the interdependent partners in the syntrophic relationship. Proteomic analysis also indicated that biosynthetic functions of *M. thermautotrophicus* are down-regulated during coculture with *S. lipocalidus* where levels of proteins involved in carbon fixation, amino acid biosynthesis, and RNA/DNA metabolism were decreased [43]. The possibility of interspecies carbon transfer was not discussed, and it is suggested that growth of *M. thermautotrophicus* is restrained in coculture. Interestingly, the *α*-subunit of the proteasome of *M. thermautotrophicus* was N-acylated during coculture which suggests that modification of global protein turnover dynamics is an adaptation to coculture [43].

In another case of methanogen-bacteria syntrophy between *Pelotomaculum thermopropionicum* and *M. thermautotrophicus*, it was shown that physical contact was key for the syntrophic interaction and that this interaction was mediated by a flagellar cap protein, FliD, where the FliD of *P. thermopropionicum* bound to *M. thermautotrophicus* [44]. Transcriptome analysis indicated that in the presence of FliD, *M. thermautotrophicus* had higher transcript levels of genes associated with methanogenesis, ATP synthesis, and hydrogenases. This is evidence of *pili*-mediated signaling involved in the onset of syntrophic interactions. Cell surface components are important in survival and establishment of relationships with bacterial partners within the gastrointestinal environment. Different *M. smithii *strains exhibited strain-specific differences in the transcript levels of their repertoire of adhesin-like proteins [28]. A qRT-PCR approach also indicated that cell surface components such as glycans and adhesin-like proteins were important in host colonization and in the establishment of syntrophic relationships in *M. smithii* [45]. The several examples presented in this section show that the consequences of syntrophic interactions amongst methanogens and bacterial partners can be significant by directly or indirectly regulating the activity of methanogens, hence deserving further attention for future studies.

Other studies have aimed to investigate the ecological dynamics of methanogens by analyzing the transcriptome of environmental samples. It was found that the *mcr*A transcript/gene ratio correlated weakly (regression coefficient = 0.76) with the CH_4_ flux from a peat soil [46]. In another study, it was shown that *mcr*A transcript levels had a positive relationship with CH_4_ flux in a CH_4_-emitting peat soil site while the transcript levels of particulate CH_4_ monooxygenase (a key gene in CH_4_ oxidation) had a negative relationship with CH_4_ flux at a CH_4_-oxidizing site [47]. In the CH_4_-oxidizing site, *mcr*A transcript levels had no correlation with CH_4_ flux [47]. Monitoring the transcript levels of key genes involved in CH_4_ flux, although informative to a small extent, is not sufficient to adequately predict CH_4_ flux dynamics. Methanogens are complex biological systems that interact with a wide variety of other microorganisms within a complex food web. Community level high-throughput sequence analysis has the potential to characterize the genetic potential, transcriptional activity (depending on whether it is the DNA or RNA that is sequenced), and diversity and abundance of microorganisms in biogas-producing microbial consortia [48]. The challenge, however, remains in interpreting such a large quantity of data in order to predict nutrient fluxes and responses to perturbations made to the system. To meet this challenge, greater knowledge is required in systems biology, at the level of microorganisms' cells and at the level of microbial communities. A significant milestone towards this goal will be the elucidation of the factors that regulate translation of mRNA, since transcript abundance alone is not sufficient to predict activity.

## 3. From Transcriptome to Phenotype

The synthesis of mRNA is merely the first step in gene expression. After transcription, the mRNA must be translated to form a protein product, and, following this, various post-translational regulatory events may alter the activity of proteins. It is a common biological phenomenon that the absolute levels of transcript abundances are poor indicators of protein levels [49, 50]. Rather, other attributes, including codon bias, gene ontology, and coding sequence length are better predictors of protein abundance [51, 52].

However, it is when comparing global responses of an organism to two or more test conditions that transcriptome studies identify profiles of differentially expressed targets in good approximation to that obtained using other “omics” technologies. For instance, reasonably good correlations are observed between differential transcript abundance and differential protein abundance for wild type *M. maripaludis* versus a mutant deficient in Ehb hydrogenase activity [50], for acetate versus methanol grown *M. acetivorans* [10] and for *M. burtonii* grown at 4°C versus 23°C [33]. Thus, methanogens use the regulation of transcript abundance as a major point of gene regulation in response to the environment. In cases where relative changes in transcript levels disagree with proteomics data in such comparisons, there is the possibility that a post-transcriptional mechanism of gene regulation is being employed. For instance, differences between transcriptomics and proteomics data of *M. burtonii* indicated that 16 genes encoding ribosomal proteins and 10 genes involved in methanogenesis were posttranscriptionally regulated in response to changes in incubation temperature [33]. Also, comparison of *M. maripaludis* transcriptome data with measurements of cellular pools of amino acids indicated that post-transcriptional regulation plays a major role in branched-chain amino acid biosynthesis [24]. Such multi-omics approaches can yield hypotheses regarding the role of posttranscriptional regulation in adapting to the conditions tested and stimulate further studies focused on particular regulatory mechanisms. Care must be taken, however, to account for the technical limitations of both transcriptomic and proteomic technologies and to account for the fact that mRNA generally has a short half-life relative to proteins.

Traditionally, transcriptome analyses, particularly those utilizing microarray technology, focus on evaluating the transcript abundance of coding sequences, although other applications exist. However, transcriptome sequencing studies are now becoming more prevalent. In comparison with microarray technology, transcriptome sequencing has greater dynamic range and is more suitable for mapping transcription start sites (TSSs) and detecting other unknown transcripts that may either be unannotated coding sequences or small RNAs (sRNAs) [53, 54]. Deep sequencing transcriptome analyses allow the identification of RNA degradation hotspots, facilitating the prediction of sequence motifs associated with RNA destabilization [55]. Information on TSSs and sRNAs will be instrumental in furthering the understanding of the role of RNA regulation in biological systems since RNA plays other crucial, though less documented roles in regulating gene expression. sRNAs may interact with mRNAs causing up- or downregulation of translation or altering the rate of mRNA turnover. sRNAs may also interact with proteins and modify their activities [56]. The ability to document a very large portion of TSSs for an organism will greatly aid investigations into how 5′- and 3′-untranslated regions (UTRs) influence RNA structure, stability, and translation.

Transcriptome sequencing of *M. mazei* mapped 876 TSSs, 208 sRNAs, and 40 small open reading frames of less than 31 amino acids [57]. This also led to the observation that *M. mazei* features long 5′ UTRs. Most of the discovered sRNAs in the transcriptome sequence analysis of *M. mazei* were located in intergenic regions, although some sRNAs were antisense to mRNAs [57]. The presence of 135 of the sRNAs depended upon nitrogen availability indicating that they play a regulatory role in nitrogen metabolism of *M. mazei*. One of the sRNAs, designated sRNA_154_, was subsequently shown to be important for optimal growth rate in nitrogen fixation conditions [58]. This indicates that sRNAs play an important role in posttranscriptional regulation of nitrogen metabolism in *M. mazei*.

Anther of the sRNAs discovered in *M. mazei*, designated sRNA_162_, was studied in greater detail [59]. Transcriptome analysis of wild type *M. mazei* versus an sRNA_162_ overexpressing derivative revealed that transcript levels of 185 open reading frames (ORFs) were differentially regulated including 48 ORFs involved in metabolism [59]. It was shown that sRNA_162_ binds to the 5′ UTR of the MM2241 transcript. By doing so, it masked the ribosome-binding site and caused translational level regulation of MM2241 which is postulated to encode a product involved in transcriptional repression of genes involved in MMA utilization. The sRNA_162_ overexpressing *M. mazei* strain adapted from growth on methanol to growth on TMA faster than the wild type strain did, further exemplifying its role as a regulator of methanogenesis.

 The aforementioned discovery of long 5′ UTRs in *M. mazei* [57] is highly significant since these are involved in the regulation of translation rate and transcript stability through various different mechanisms [60]. A 5′ UTR was important in the methanogenic substrate-dependent regulation of CODH/ACS activity in *Methanosarcina* spp. by an uncharacterized mechanism that was likely either early transcriptional termination or endoribonuclease activity against the 5′ UTR [61]. In *M. acetivorans*, 5′ UTRs are also known to be involved in regulating the expression of different isozymes of methanol specific methyltransferases [62].

Bioinformatics work previously inferred that methanogens generally carry 5′ UTRs while other groups of *Archaea* often feature leaderless mRNAs [63]. Since then it was shown that haloarchaeal transcripts are mostly leaderless [64], and a recent transcriptome sequencing study showed that most *Sulfolobus solfataricus* mRNAs completely lack 5′ UTRs [55]. In *S. solfataricus*, leadered mRNAs required Shine-Dalgarno (SD) motifs to direct the 30S ribosome subunit to the translation initiation region, while correct positioning of the 30S subunit on leaderless mRNAs required a prebound initiator tRNA [65]. It is, however, worth noting that 70S ribosomes bind with higher affinity to leaderless mRNA than do 30S ribosomal subunits [66]. It was also suggested that SD-dependent initiation would operate during the translation of distal cistrons of polycistronic mRNAs [65]. However, the 5′ UTRs of most leadered haloarchaeal transcripts lacked an SD motif [64]. It is significant that methanogens may generally feature 5′ UTRs while most transcripts within the Haloarchaea and Crenarchaea lack 5′ UTRs and that the Haloarchaea and Crenarchaea seem to differ in their requirement for SD motifs in their 5′ UTR-containing mRNAs. Thus, it appears that there are differences in the mechanisms of translation initiation between these three groups of *Archaea*.

Further evidence of key differences in posttranscriptional control within the *Archaea* concerns 3′ UTRs. Polyadenylation at the 3′ end of mRNAs influenced RNA degradation in hyperthermophilic *Archaea *and in some methanogens but not in other methanogens nor the Haloarchaea [67]. Most of what is known about the role of 3′ UTRs in *Archaea* comes from the study of nonmethanogenic *Archaea*. For instance, the simultaneous presence of both the 5′ and the 3′ UTRs was shown to be required for translational regulation for two genes of *Haloferax volcanii* [68]. It was the 3′ UTR that dictated the direction of translational regulation [68], though this regulatory mechanism remains uncharacterized.

Differences in posttranscriptional control between different archaeal groups are significant when it is considered that methanogens are generally found to be difficult to manipulate in the laboratory for biochemical studies due to their high oxygen sensitivity. Consequently, other archaeal systems are often used as models to investigate a variety of fundamental processes, including translation related processes. However, owing to differences in posttranscriptional control and mRNA features between different archaeal groups, the use of methanogen models must be considered. The roles of 5′ UTRs and SD motifs in regulating the translation of methanogen mRNAs need to be clarified in future studies. The roles of the 3′ UTRs in methanogenic *Archaea* are also unclear. Current sequence annotation methods are insufficient to reliably predict TSSs, small open reading frames, and sRNAs. However, sequence annotation methods will likely improve due to transcriptome-sequencing work providing a wealth of training sets for the development of new bioinformatic tool sets. These advances will likely be very influential in ongoing research into methanogens and biological systems in general. Ultimately, the paucity of experimental data related to RNA regulation of translation in the *Archaea* must be addressed.
